# Supplementary material for: Divergence of the PIERCE1 expression between mice and humans as a p53 target gene
Source: PLoS One. 2020 Aug 3;15(8):e0236881. doi: 10.1371/journal.pone.0236881 (PMC7398528; doi:10.1371/journal.pone.0236881)
Supplement: S1 Table — (DOCX) [file pone.0236881.s005.docx]

**Supplementary Table 1. Primer sets for qPCR**

| **Primer** | **Sequence** |
| --- | --- |
| mGapdh – F | AGG TCG GTG TGA ACG GAT TTG |
| mGapdh – R | GGG GTC GTT GAT GGC AAC A |
| hGAPDH – F | GGT GAA GGT CGG AGT CAA CG |
| hGAPDH - R | TCA TTG ATG GCA ACA ATA TC |
| mp53 – F | CCC CTG TCA TCT TTT GTC CCT |
| mp53 - R | AGC TGG CAG AAT AGC TTA TTG AG |
| mp21 - F | CCT GGT GAT GTC CGA CCT G |
| mp21 - R | CCA TGA GCG CAT CGC AAT C |
| mPierce1 – F | CAG AGA AGA CCA GCG ACT AC |
| mPierce1 – R | TGT ACA TGG AAA CAG CTT CC |
| hp53 - F | ATG GAG GAG CCG CAG TCA GAT |
| hp53 - R | GCA GCG CCT CAC AAC CTC CGT |
| hp21 - F | AGG TGG ACC TGG AGA CTC TCA G |
| hp21 – R | TCC TCT TGG AGA AGA TCA GCC G |
| hPIERCE1 – F | ACA GGA CCA GTA ACC AGG CT |
| hPIERCE1 – R | CGC TGC AAG TTG TTG GGA AA |
| hBAX – F | TCA GGA TGC GTC CAC CAA GAA G |
| hBAX – R | TGT GTC CAC GGC GGC AAT CAT C |
| hMDM2 - F | TGT TTG GCG TGC CAA GCT TCT C |
| hMDM2 – R | CAC AGA TGT ACC TGA GTC CGA TG |
| hPAI – F | CTC ATC AGC CAC TGG AAA GGC A |
| hPAI - R | GAC TCG TGA AGT CAG CCT GAA AC |
