# Supplementary material for: Divergence of the PIERCE1 expression between mice and humans as a p53 target gene
Source: PLoS One. 2020 Aug 3;15(8):e0236881. doi: 10.1371/journal.pone.0236881 (PMC7398528; doi:10.1371/journal.pone.0236881)
Supplement: S2 Table — (DOCX) [file pone.0236881.s006.docx]

**Supplementary Table 2. siRNA sequences for p53 knockdown**

| **siRNA** | **Sequence** |
| --- | --- |
| siCTRL | UUC UCC GAA CGU GUC ACG UdTdT  ACG UGA CAC GUU CGG AGA AdTdT |
| sip53 #1 | CUA CUU CCU GAA AAC AAC GdTdT  CGU UGU UUU CAG GAA GUA GdTdT |
| sip53 #2 | GGA CAU ACC AGC UUA GAU UdTdT  AAU CUA AGC UGG UAU GUC CdTdT |
| sip53 #3 | UGU GAG GGU UAA UGA AAU AdTdT  UAU UUC AUU AAC CCU CAC AdTdT |
| sip53 #4 | CCC UGU CUG ACA ACC UCU UdTdT  AAG AGG UUG UCA GAC AGG GdTdT |
